# Supplementary material for: Are carbon emissions trading and green financial instruments synergistic? -Comprehensive quantitative research based on content analysis
Source: PLoS One. 2024 Mar 7;19(3):e0298601. doi: 10.1371/journal.pone.0298601 (PMC10919601; doi:10.1371/journal.pone.0298601)
Supplement: S1 Data — (ZIP) [file pone.0298601.s001.zip › Data/地级市Z_F工作报告.zip!/║■▒▒/╛ú╓▌╩╨/└┤╫╘╣1⁄2╓┌║┼í╛┬φ┐╦╩2╛▌═°í┐_2015.docx]

各位代表：

现在，我代表市人民政府向大会报告工作，请予审议，并请各位政协委员和列席人员提出意见。

一、2014年工作回顾

过去的一年，是全面深化改革的开局之年，是荆州经济增量提质、社会全面进步之年。面对宏观经济持续下行压力，在省委、省政府和市委的正确领导下，在市人大、市政协的监督支持下，我们紧紧团结和依靠全市人民，深入实施壮腰工程，扎实做好各方面工作，主要经济指标增速高于全国、快于全省。地区生产总值1480.5亿元、增长9.8%，三次产业结构为23.4：44.6：32；固定资产投资1571.1亿元、增长22%；社会消费品零售总额831.4亿元、增长13.3%；出口总额13亿美元、增长15%；地方公共财政预算收入88.2亿元、增长22.5%；城镇常住居民人均可支配收入23128元、增长9.8%，农村常住居民人均可支配收入12625元、增长11.9%；年度节能减排任务圆满完成。

（一）坚定不移壮产业，经济总量进一步扩大。

工业经济质效提升。规模以上工业增加值增长11.1%，增幅连续24个月居全省第一方阵，实现利润118.1亿元、增长14.1%。规模以上工业企业净增110家、达到1110家；销售收入10亿元以上工业企业新增4家、达到29家，纳税亿元以上工业企业新增1家、达到6家。华讯方舟、伟特电子、高田汽配、中联重科、中纺油脂、天银循环、白云边扩能、长江医药、祥锦汽配、华中玻铝、长利玻璃等项目进展顺利，装备制造、医药化工、电子信息分别实现主营业务收入500亿元、260亿元和135亿元。监利-江陵家纺产业新纳入省重点扶持成长型产业集群。

农业发展活力增强。全市农业增加值增长5.2%，农产品加工业产值突破1200亿元，农产品加工业产值与农业总产值之比达到2∶1。粮食新增产量、新型农业经营主体总量居全省市州之首，淡水产品、油菜籽产量继续排名全国市州之首。监利县、公安县、洪湖市荣获“全国产粮大县”称号；监利水稻工厂化育秧模式领先全国。全市争取高标准基本农田土地整理项目资金8亿元，建设规模53万亩，居全省第一位。

第三产业持续增长。商贸物流业繁荣活跃，限额以上商贸企业新增357家、达到1027家。电子商务、网络消费等新型业态发展迅猛，快递业务量、电商交易额分别增长92%和58%，两湖绿谷电商销售突破140亿元；万达广场、绿地之窗、义乌小商品城等城市综合体建成营业，催生了荆州商业新格局。全市港口年吞吐量2850万吨、增长8.9%。文化旅游业稳步发展，荆州古城环城旅游景区规划编制完成，纪南生态文化旅游区纳入省级战略实施，熊家冢国家考古遗址公园、关公文化园、九老仙都等景点景区建设加快，全市旅游总收入137.7亿元、增长23%。金融生态环境持续向好，连续7年被评为省“A级信用市州”；新增贷款173.2亿元，增量居全省第三位；多层次资本市场建设取得实质性进展，菲利华在深圳创业板上市，能特科技资产以定向增发方式整体上市，金科环保在“新三板”挂牌，打破近13年来没有企业上市的局面。

（二）持之以恒抓项目，发展基础进一步稳固。

重大基础设施建设加快。蒙华铁路可研报告、荆州机场选址和华电江陵电厂项目得到国家批复，沿江铁路、荆沙铁路扩能改造、江汉平原货运铁路前期工作顺利推进，石首长江公路大桥、沙公高速开工在即，荆州长江公铁两用桥、荆江航道整治进展较快，引江济汉工程通水通航。江北、洪监等6条高速公路同步施工，江南高速建成通车，结束了松滋、石首无高速公路的历史；荆松一级公路建设加快，荆州区旅游公路、318国道荆州段改扩建项目启动建设，完成交通投资132.5亿元，超过“十一五”时期交通投资总和。荆江大堤综合整治、荆南四河堤防加固等水利工程投资21.7亿元，电力建设投资5.3亿元，均为近年来新高。第四代移动通信设施建设步伐加快，信号覆盖面走在全省前列。

园区建设力度较大。全市开发区、产业园区新拓展面积40平方公里，基础设施建设投入66亿元，入园企业新增232家。荆州开发区规模以上工业企业实现利润24亿元、增长50%，上海大道、曙光大道建成通车，“三纵七横”路网格局更加完善；华中农高区获批国家农业科技创新与集成示范基地，14个产业和基础设施项目同步推进，太湖大道、农高大道建成通车；纪南生态文化旅游区建设稳步推进，楚都大道、凤凰大道开工建设。洪湖市与武汉经济技术开发区合作共建新滩工业园，荆州区九阳机械电子工业园、沙市区锣场工业园、江陵县沿江产业园、松滋市临港工业园、公安县青吉工业园、石首市金平工业园、监利县城东工业园基础设施建设和项目入驻步伐加快。

招商引资突破千亿。全年实际到位资金1040亿元、增长40.5%。新批外商投资企业9家，实际利用外资1.2亿美元、增长12.7%。全市引进亿元以上项目255个，其中工业项目237个，荣成纸业、昌泰纸业、中航粮油、琅日纤维、腾国迅硅能电池等一批重大项目落户荆州，通宇通讯、荆龙汽配、顺昌科技、宝龙纺织等21个项目当年签约落户并建成投产。

（三）借力省运建城市，城乡面貌进一步改观。

省运会承办精彩圆满。我们举全市之力，精心承办湖北省第十四届运动会，赛事活动特色鲜明、安全节俭、隆重热烈，被省组委会授予“省运会承办突出贡献奖”、“体育道德风尚奖”。荆州市体育中心顺利建成，奥体中心、古城健身中心等场馆改造升级；3.8万人参演大型健身操“荆州动起来”，创大世界基尼斯纪录；夺得金牌282枚，创历史最好成绩。省运会激发的“自强不息、奋力拼搏、团结协作、不断超越”的竞进精神，成为全面建设荆州的宝贵财富和强大动力。

中心城区品位提升。荆北、沙北、商务等片区建设提速，大城市格局全面拉开。市政投入19.5亿元，新建改造城市道路57公里，楚源大道、明珠大道、天谷大道、凤凰路等主次干道建成通车；临江仙公园、明月公园、荆襄河湿地公园和西干渠带状公园绿地建成开放，新建城市公园绿地近100公顷。城管精细化综合考评全面推开，大城管格局基本形成。开展“三禁两治”环保行动，中心城区空气质量明显好转，工作经验全省推广；实施生态引水工程，护城河、荆沙河等水环境得到改善。

小城镇建设管理加强。松滋市、公安县成功创建省级文明城市，洪湖市成为全省首批水生态文明城市建设试点。小城镇建设管理体制改革稳步推进，荆州区弥市镇等13个乡镇成功创建国家重点镇，监利县新沟镇获评全省农村信息化建设示范镇；沙市区岑河镇、石首市新厂镇、江陵县三湖农场等11个“四化同步”发展试点镇建设加快。新农村建设力度加大，改造农村危旧房5056户，新建通乡通村公路976公里，新增农村安全饮水人口57.8万人。

（四）积极稳妥促改革，内生活力进一步增强。

各项改革不断深化。加大行政审批制度改革和简政放权力度，市级行政权力减少38.2%，行政审批事项减少20.1%，市级议事协调机构由483个精简到140个。事业单位分类改革稳步推进，中介机构清理规范基本完成，公积金管理体制、县级城管综合执法改革落实到位。农村承包土地确权登记颁证试点工作启动，江陵县整县推进工作考核位于全省前列。财税改革加快推进，中心城区财税征管体制继续完善。全市农信社改革圆满完成。医疗卫生体制改革进展顺利。食品药品监管体制改革基本完成。商事登记制度改革成效显著，市场主体新增6.3万户、增长26.8%，总量达到29.6万户。

自主创新能力提高。高新技术企业新增19家、达到89家，产值460亿元、增长16%。21项科技成果获省以上科技奖，登记技术合同交易额9.8亿元。中油科昊等5家企业获批省级工程技术研究中心，索瑞电气等8家企业成为省知识产权示范企业试点。新设院士专家工作站13家，组建了水产技术创新战略联盟。新增中国驰名商标2件、国家地理标志保护产品和地理标志证明商标7件，新增注册商标1307件、增量居全省第二。

（五）千方百计惠民生，居民生活进一步改善。

基本民生得到保障。政府民生性支出218.6亿元，占地方公共财政预算支出的79%。十件实事全面完成。全市新增就业人员8.9万人，扶持创业6288人，带动就业2.4万人。保障性住房开工35963套，基本建成19038套。落实企业退休人员基本养老金调整政策，人均养老金水平达到每月1566元。新建城镇社区居家养老服务中心25个、农村互助照料活动中心290个。中心城区低保标准由每月390元提高到430元，农村低保标准由每年1900元提高到2500元。基层医疗卫生服务和重大疫病防控不断加强，在册晚血病人得到免费救治。

社会事业全面发展。县域义务教育均衡发展，洪湖市通过教育部评估验收，沙市区、公安县通过省级验收；成功组建荆州职业教育集团。文化广播电视事业蓬勃发展，在全省率先建成农村智能广播网；“石首跳三鼓”纳入国家级非遗代表性项目名录。洪湖湿地获批国家级自然保护区，环荆州古城湿地公园、公安崇湖湿地公园纳入国家级湿地公园建设。开展“绿满荆州”行动，植树造林37.6万亩。计划生育政策较好落实，出生人口政策符合率93.5%。文明创建活动深入人心。妇女儿童、老年人和残疾人合法权益得到保护。我市荣获“第三次全国经济普查先进集体”称号。国防动员、国防教育、人民防空、民兵预备役和双拥工作得到加强。外事侨务、台湾事务、民族宗教、防震减灾、对口支援等工作取得新成绩，机关事务管理、物价、扶贫、慈善、农垦、移民、供销、烟草、盐务、气象、档案、社科、史志、保密和无线电等工作取得新进步。

社会治理能力不断提升。平安法治建设强力推进，综治信访维稳成效明显，社会治安大局持续稳定。网格化管理实现城乡全覆盖，立体化治安防控体系基本建立，中心城区抢劫、抢夺、扒窃案件大幅下降，公众安全感、治安满意度和执法满意度明显提升。松滋市被评为全国“六五”普法先进县市。积极受理群众来信来访，累计化解各类信访积案432件。安全生产形势保持稳定，安全事故起数、死亡人数、受伤人数和直接经济损失分别下降6.9%、7.8%、19.6%和7.7%。

（六）严肃认真反四风，政府自身建设进一步加强。

作风建设成效显著。深入开展党的群众路线教育实践活动，聚焦“四风”查问题、抓整改，清理“吃空饷”4417人，调减超标办公用房4.7万平方米，“三公”经费比上年减少26.5%；公款吃喝、请客送礼、公车私用等现象有效遏制，群众反映突出的“中梗阻”、政务服务效率不高等问题明显好转，一批违反八项规定精神和违纪违法案件得到查处。

依法行政全面推进。严格遵守和执行各项法律法规，自觉接受人大的法律监督、工作监督和政协的民主监督，积极回应民主党派、工商联、无党派人士和人民团体对社会热点问题的关注，及时办结省级建议提案17件、市人大代表建议96件、市政协提案337件。政务督查、政务公开、应急管理工作得到加强，公众诉求得到及时处理。强化行政执法监督、审计监督和审后整改，行政行为更加透明规范。

各位代表！过去一年，是壮腰工程“三年见成效”之年。三年来，全市上下围绕发展抓工业、围绕工业抓招商、围绕招商抓环境、围绕环境抓问责，全力推进“五路壮腰”，实现了“三个翻番”，收获了“四个千亿”，取得了“六个提升”。即固定资产投资、规模以上工业企业销售收入、地方公共财政预算收入实现三年翻一番；地区生产总值、固定资产投资、农产品加工业产值、招商引资实际到位资金相继突破千亿元；地区生产总值、固定资产投资、规模以上工业增加值、社会消费品零售总额、地方公共财政预算收入、城镇常住居民人均可支配收入等主要经济指标增幅位次逐年提升，进入全省第一方阵。可以说，壮腰工程阶段性目标任务的顺利完成，为“五年大跨越”、“十年大振兴”奠定了重要宝贵的基础，也更加坚定了我们竞进新目标的信心和决心。

各位代表！上述成绩的取得，是省委、省政府和市委正确领导、科学决策的结果，是市人大、市政协有效监督、鼎力支持的结果，是全市人民同心同德、克难奋进的结果。在此，我代表市人民政府，向全市广大干部群众，向人大代表、政协委员，向各民主党派、工商联、无党派人士、各人民团体，向驻荆部队、武警部队，向所有关心支持荆州经济社会发展的各界人士，表示衷心的感谢并致以崇高的敬意！

各位代表！在肯定成绩的同时，我们也清醒地认识到，政府工作仍然面临不少困难和挑战：地区生产总值、规模以上工业增加值、社会消费品零售总额增速趋缓，经济较快发展的压力增大；经济总量不大，二、三产业发展不够，工业企业尤其是中小微企业融资难、融资贵问题依然十分突出，现代服务业尤其是文化旅游业资源优势没有得到充分发挥；地方财力仍较薄弱，基本公共服务质量和水平不高，社会保障、城市管理、食品安全、环境保护等方面群众不满意的问题依然较多；各级政府及其工作人员在思想观念、工作方法、工作作风等方面与人民群众期待还有一定差距。对此，我们一定更加重视，认真加以解决。

二、2015年工作任务

2015年，是全面深化改革的关键之年，是全面推进依法治国的开局之年，也是“十二五”规划的收官之年。

当前，我国经济已经进入了调速换挡、转型升级的新常态，我们要深刻认识、积极应对。新常态带来新挑战，必须坚定信心、保持定力。既要看到新常态条件下可能面临的不利形势，切实增强危机意识、忧患意识，更要看到国内经济总体向好的基本面没有变，荆州振兴的良好态势没有变。只要我们稳住心神、理性对待，就一定能适应新常态、实现新发展。新常态蕴含新机遇，必须牢牢把握、乘势而上。中央高度重视新常态背景下区域经济发展和差异化宏观政策调控，大力推进长江经济带发展和洞庭湖生态经济区建设；省委、省政府重点支持纪南生态文化旅游区和江汉运河生态文化旅游城镇带建设，有利于我们争取重大项目和政策扶持。只要我们深度谋划、因势利导，就一定能发展超常规、赢得新优势。新常态要有新作为，必须改革创新、精准发力。新常态倒逼经济结构调整，表面看是发展调速换挡，本质上是发展方式转型、体制机制再造，是对深化改革、创新发展的新要求和新定位。只要我们找准改革创新的结合点和着力点，就一定能实现新突破、开创新局面。

今年政府工作的指导思想是：深入贯彻落实党的十八大、十八届三中四中全会和习近平总书记系列重要讲话精神，认真落实中央、省委重大决策和市委工作部署，坚持“稳中求进”总基调和“竞进提质、升级增效”总要求，主动适应经济发展新常态，更加注重产业增量提质，更加注重改革创新驱动，更加注重生态文明建设，更加注重保障和改善民生，努力实现经济持续健康发展和社会和谐稳定，为壮腰工程“五年大跨越”奠定坚实基础。

今年经济社会发展的预期目标是：地区生产总值增长9.5%，固定资产投资增长20%以上，社会消费品零售总额增长13%，外贸出口增长11%，地方公共财政预算收入增长14.5%，城镇常住居民人均可支配收入增长9.5%，农村常住居民人均可支配收入增长10%，居民消费价格涨幅控制在3.5%以内，城镇登记失业率控制在4.5%以内，人口自然增长率控制在6‰以内，完成省下达的节能减排任务。

今年，我们将认真做好以下八个方面的工作：

（一）做大做强工业经济。依托创新驱动，推进“两化融合”，优化存量、扩大增量、提升质量，加快新型工业化进程，力争规模以上工业增加值增长11%。

调整优化工业结构。积极争取省政府产业基金、产业集群专项资金和中小企业发展基金，支持传统产业加快技改扩能、提升装备技术水平，推动装备制造、医药化工、电子信息等产业规模化、高端化发展，打造荆州支柱产业升级版。继续抓好华讯方舟、长江液晶、同洲电子、江汉建机、怡德石油、美帆金属、松滋水泥、荣成纸业、昌泰纸业、瀚煜建材、玉沙纺织、雪花秀化妆品等145个工业和技改项目。积极承接产业转移，抢前布局新兴产业，加强与“核高基”、新一代宽带天线移动通信、重大基础装备等重大科技专项的对接，引进培育一批具有核心技术、引领性强的产业和企业。

培育壮大市场主体。坚持企业需求导向，落实和完善土地、融资、人才等扶持政策，统筹安排重大产业发展基金、生产调度资金等政府性资金，支持白云边、沙隆达、楚源、四机厂、美的、恒隆、法雷奥、凯乐、奥达、金安、广兴通信等一批骨干企业和行业龙头扩规模、上档次，带动优势产业链式发展、抱团发展。继续实施中小企业成长工程，探索设立企业续贷周转金，重点支持成长型、科技型中小微企业“小进规”、“规改股”、“股上市”，力争新增规模以上企业100家以上。继续实施商标战略和质量振兴战略，鼓励企业加大自主创新投入，争创精品名牌、抢占细分市场。继续实施“双百企业家培训计划”，加快培养一支有战略眼光、创新能力和社会责任的企业家队伍。

提升园区发展水平。充分发挥开发区工业“主战场”作用，支持开发区和工业园区完善配套功能，增强承载能力，实现内涵式发展。支持设立创业投资引导基金，支持开发区与高等院校、科研机构共建研发平台和创新战略联盟，促进科技成果加速转化。突出园区产业定位，规范项目落户优惠政策，鼓励建设通用厂房，引导关联产业、企业和项目向园区集聚，进一步优化布局，提高投入产出比。

（二）转变农业发展方式。坚持农业产业化、规模化、品牌化并重，走产出高效、产品安全、资源节约、环境友好的现代农业发展之路。

大力发展效益农业。遵循市场规律，推进农业结构调整，着力提高农业发展质量和效益。加大投入力度，加强农田水利、中小泵站、土地整理等基础设施建设，推广工厂化育秧、机械化插秧，提高粮食综合生产能力，积极创建“百亿斤粮食大市”。做大畜禽养殖、名优水产、特色果蔬等产业板块，提高规模化、集约化水平。强化农产品生产源头治理和产销全程监管，保障农产品质量安全。支持华中农高区打造一、二、三产业融合发展的现代农业示范区，力争今年完成基础设施和产业投资50亿元，为创建国家级农业高新区打好基础。

加快打造农业全产业链。坚持用全产业链的思路谋划农业，延长产业链，锻粗价值链，重点打造以福娃集团、宏凯油脂为龙头的粮油全产业链，以襄大农牧、温氏集团为龙头的畜禽全产业链，以德炎水产、洪湖清水为龙头的水产加工全产业链和以拍马纸业、吉象木业为龙头的林纸加工全产业链。继续实施“四个一批”工程，支持监利县、洪湖市建成百亿元农产品加工园，支持龙头企业壮大实力、建设百亿企业，力争全市农产品加工业产值新增200亿元以上。

培育新型农业经营主体。认真开展农村土地承包经营权确权登记颁证工作，办好农村集体土地“三权分置”改革试点，引导农村土地向专业大户、家庭农场、农民合作社、农业企业流转，力争全市新型农业经营主体经营面积增长32%、达到200万亩。加大市场准入、资金支持等方面的扶持力度，支持农业新型市场主体做大做强、提升档次、规范发展，加快构建专业合作、供销合作、信用合作“三位一体”的农村新型合作体系。落实各项强农惠农政策，确保农民持续增收。

（三）加快现代服务业发展。主动顺应个性化、多样化消费需求，提升消费水平，拓宽消费领域，促进现代服务业提质增效。

突破性发展文化旅游业。坚持走多层次、复合型旅游发展之路，加大政策支持力度，加强旅游产品打造和功能配套，加快把文化旅游业培育成战略性支柱产业。启动旅游总体规划修编，重点打造纪南生态文化旅游区和关公文化园，着力推动荆州古城历史文化景区和洪湖旅游景区提档升级、争创5A级景区，统筹推进江汉运河生态景观带、松滋洈水、石首天鹅洲、公安黄山头、监利周老嘴等景点景区建设。完善精品旅游线路，抓紧建成荆州游客集散中心，推动市域旅游板块串连互通、资源整合。支持太湖桃花村建设国家级休闲农业示范点，加快建成一批旅游名镇名街名村。多形式开展宣传推介和市场营销，力争旅游总收入增长20%以上。

大力发展商贸物流业。认真对接和实施服务业“五个一百”工程，充分发挥大型商贸流通企业、城市综合体、城市商圈的带动作用，提升商贸服务业品位和档次。建立健全农村商贸网点和配送体系，改善消费环境，拓展消费市场。支持快递企业延伸业务、健全网络，引进和培育电商市场主体，鼓励生产企业和传统商贸流通企业加强电子商务应用，力争快递业务和电商交易额增长45%以上。推动中心城区物流业集聚发展，培育一批具有辐射力的专业市场和物流园区，加快构建现代物流基地。

培育发展新型业态。围绕服务创新、业态创新，培育消费热点，释放消费潜力。积极发展社区服务、健康养老、体育健身、文化娱乐、旅游休闲等服务业态，不断满足居民多层次消费需求。大力发展工业设计、技术服务、商务咨询、节能环保、检验检测等生产性服务业，推动服务业与制造业深度融合。落实新型服务业发展扶持政策，不断增强消费对经济增长的拉动力。

（四）深入推进改革开放。坚持改革不停顿、开放不止步，以改革增动力、增活力，以开放促改革、促发展。

全面深化改革。加快推进简政放权，实施市县行政审批通用目录制度，推行网上联审联办，努力形成有利于大众创业、万众创新的体制机制和政策环境。扎实做好市县政府机构改革工作，完善事业单位分类改革，完成公务用车改革。推进财税体制改革，重点做好中期财政规划编制、预算编制、政府购买社会服务和新一轮“营改增”等工作。深化国企改革，支持投融资平台完善功能、做大做强，支持经营性事业单位转企改制为市场主体。鼓励和促进非公有制经济健康快速发展。深化国有农场改革。启动不动产统一登记工作。加快医疗卫生体制改革，稳步推进机关事业单位养老保险制度改革。

强化招商引资。继续开展产业链招商、驻点招商、以商招商，瞄准长三角、珠三角、京津冀等区域和国内外500强企业、行业龙头企业，着力引进一批规模较大、带动力强、集群效应明显的优质企业和项目，力争招商引资实际到位资金增长25%。统筹推进市区、市县联动招商，促进同质集聚、合理布局、错位发展。创新招商方式方法，更加注重为招商项目营造公平环境、提供优质服务。

积极开展区域合作。抢抓国家扩大内陆开放新机遇，主动对接长江经济带和洞庭湖生态经济区战略，找准定位、深度融入，致力打造长江经济带重要节点城市。谋划和推进铁路、公路、港口等基础设施在荆州境内互联互通，尽快建成区域性综合立体交通枢纽。加强产业、生态等资源要素对接，积极参与区域经济合作，推进产业差异发展、协作配套，在更广范围利用发展资源、拓展经济腹地。加强大通关建设，拓展口岸功能，扩大优势产品出口规模，引导和鼓励有实力的企业参与国际市场竞争，不断提升开放性经济发展水平。

（五）突出项目投资带动作用。把握全面深化改革带来的增长动力和新型城镇化带来的增长潜力，以大投入聚活力、增后劲、促发展。

科学编制“十三五”规划。统筹做好“十三五”规划与专项规划编制工作，增强规划的前瞻性、指导性。紧紧围绕宏观政策和重大发展战略，策划一批交通、水利、产业、生态环保、粮食安全和民生改善项目。抓紧推进项目前期工作，力争沿江铁路、江汉平原货运铁路、荆州机场、四湖流域综合治理、江陵煤制气等重大项目纳入国家和省规划，确保项目储备充实、接替有序、支撑有力。

加快实施重大基础设施项目。继续开展“项目建设年”活动，以交通、水利、能源等项目建设支撑投资增长。积极推进蒙华铁路对接服务工作，开工建设石首长江公路大桥、沙公高速、荆州港斗湖堤港区朱家湾综合码头二期等工程，加快推进荆州长江公铁两用桥、洪监高速、江北高速、荆松一级公路、荆州区旅游公路和318国道荆州段改扩建工程，确保潜石高速江陵段、东卷高速、武汉城市圈环线荆州段建成通车。加快实施荆江大堤综合整治、荆南四河堤防加固、四湖流域综合治理和洪湖分蓄洪区东分块蓄洪工程等项目建设，完成荆江航道整治主体工程。尽早开工建设华电江陵电厂和煤炭储配基地。

着力推进项目落地。完善联合审批、联席会议制度，探索项目立项和规划设计联审联办，提高项目审批效率。继续实施重点项目“秘书制”服务和工业项目全程代办，为项目建设提供全方位、全天候、全过程服务。实施重大项目动态管理，健全项目督查工作机制，落实领导联系重大项目制度，及时协调解决征迁、用地、配套、融资等方面的问题。规范政府投资行为，提高政府投资效益。

（六）统筹城乡一体化发展。注重规划引领，强化生态保护，推进城乡共建，力争城镇化率突破50%。

加快中心城区建设发展。积极争创国家卫生城市、国家生态园林城市、国家环保模范城市、国家森林城市和全国文明城市，持续优化城市人居环境。启动城市总规修编，建立规划督察制度，重点整治社区违法建设行为。继续加大城市基础设施建设力度，加快城中村、棚户区改造和保障房、还迁房建设，以片区开发带动古城人口疏散、老城改造和城市品质提升。推进新图书馆、规划档案馆建设，加快荆州中学、沙市中学、荆州市中心医院迁建。继续实施城市绿化、美化、亮化工程，重点建设荆襄河湿地公园二期、范家渊公园和玉桥公园，打造环古城景观带和荆江风情带。加强智慧城市建设，落实“大城管”体制，推进城市管理信息化、精细化。

提高城镇化水平。按照“四化同步”要求，坚持以人为本、产城融合，推进“多规合一”，统筹城乡建设、产业发展和人口布局，推进县城、重点镇和新农村建设，构建新型城乡发展格局。支持县市根据主体功能区规划，壮大优势产业、特色产业，增强县域经济核心竞争力和综合实力。支持中心镇、口子镇、特色镇发展，加快打造一批以产业为支撑的工业强镇、商贸重镇。积极开展文明卫生城镇创建活动，全面提升城镇管理水平。落实户籍相关政策，推动基本公共服务、公共资源均衡配置，加快农业转移人口市民化进程。

加强生态文明建设。认真贯彻落实新环保法，坚持污染防治与生态保护并重，做到工业污染、农业面源污染、生活污染“三污同治”，结构减排、工程减排、管理减排“三管齐下”，严守生态红线，促进绿色发展。继续加强城市污水处理设施建设，提升城市生活污水处理能力；实施护城河、西干渠截污和生态补水工程，改善中心城区水生态环境。完善四湖流域乡镇污水处理厂管理、营运、维护长效机制，确保水质稳定达标排放。推进“绿满荆州”行动，力争植树造林61万亩，支持松滋市、石首市创建全省林业生态示范县市。在全市开展“三禁两治”工作，加强农村生活垃圾治理，推进环境综合整治，强化环境监管和执法，让城乡居民共享清新空气和优良生态。推进能源资源节约利用，支持重点行业、公共机构等领域节能降耗，大力发展循环经济。

（七）提高财税金融服务能力。充分发挥财政扶持作用，不断完善金融服务体系，为实体经济发展提供有力支撑。

提升财税工作水平。加大财政对重点行业、骨干企业支持力度，努力培植税源，做大财政“蛋糕”。坚持依法治税、严格税收征管，强化对重点行业、企业、税种和环节的监控，努力增加税收收入。牢固树立“公共财政、阳光财政”理念，加大对民生事业、重点领域改革和重点项目建设的支持保障力度，进一步强化财政预算执行和收支公开。注重盘活财政间歇资金，推行政府购买服务，提高财政资金使用效率。

提升金融服务水平。加大金融招商力度，积极引进股份制银行和村镇银行，创新助保贷、助农贷、仓单质押贷款等金融产品和服务方式，强化对地方经济的信贷支持，确保贷款余额过千亿，力争达到1200亿元。引导民间金融规范发展，支持大正担保等一批融资性担保机构做大做优。落实企业上市奖励政策，鼓励和引导企业通过多层次资本市场扩大直接融资，力争“新三板”挂牌企业总数突破10家，“四板”挂牌企业总数突破30家。加强社会信用体系建设，建立失信“黑名单”制度，规范保险、证券市场，严厉打击非法集资和高利贷行为，切实营造良好的金融生态环境。

（八）全面加强社会建设。着力推进以改善民生为重点的社会建设，创新社会治理，维护公平正义，确保人民安居乐业、社会安定有序。

强化基本民生保障。落实就业创业扶持政策，统筹做好城乡劳动者就业和企业用工服务，抓好大中专毕业生、复员退伍军人、城镇新增劳动力和农村转移劳动力的就业指导，重点帮扶零就业家庭、残疾人、低保对象和下岗失业人员。加强社会保险扩面征缴，全面推进被征地农民养老保险工作。着力推进社区居家养老和农村互助养老，大力支持多元化社会养老服务。健全农村留守儿童、妇女、老人关爱服务体系，发展社会福利、慈善和残疾人事业。完善住房公积金提取、使用和监管机制。强化专项扶贫项目监管，推进科学扶贫、精准扶贫。落实城乡社会救助政策，稳步提高城乡低保、农村五保供养水平。扎实开展以“村村通客运”为主题的新一轮“三万”活动。

繁荣各项社会事业。重视农村学前教育，均衡发展义务教育，优化提升高中教育，加快教育信息化发展。支持中高职院校资源整合，提高特色现代职业教育发展水平。支持长江大学等在荆高校与地方共建共兴、融合发展。加强公民道德和精神文明建设，弘扬社会主义核心价值观，健全现代公共文化服务体系，注重非物质文化遗产保护与传承，促进文化大发展大繁荣。完善城乡医疗卫生服务网络，探索建立分级诊疗就医新机制。加强艾滋病、血吸虫病等重大疫病防控。严格治理出生人口性别比，促进人口长期均衡发展。加强体育健身场所建设管理，不断满足全民健身需求。坚持节约集约利用土地，大力盘活土地存量，提高用地效率。做好人口抽样调查工作。支持工会扩大工作覆盖面。认真抓好国防动员、民兵预备役和人民防空工作，支持驻荆部队、武警部队建设，进一步做好外事侨务、台湾事务、民族宗教、防震减灾、对口支援、邮政通信、移民、农垦、盐务、供销、气象、史志、保密和无线电等工作。

创新社会治理方式。扎实推进平安法治荆州建设，全面提升社会综合治理水平。规范城乡网格化管理，推动资源集成、服务下沉，充分发挥网格平台作用。加强反恐维稳、禁毒整治等工作，依法打击各类违法犯罪活动。开展“信访法治建设年”活动，健全涉法涉诉信访依法终结机制。强化安全生产红线意识和公共安全教育，严格重点行业、重点领域安全生产监管，加强农村交通安全、劳动密集型企业和人员密集场所公共安全管理，坚决遏制重特大事故发生。完善自然灾害、事故灾难的监测预警，提高应急处置、现场管理和救援保障能力。高度重视食品药品安全，深入推进餐桌污染治理，建设食品放心工程。整治和规范农资、卷烟、成品油等市场秩序，严厉打击假冒伪劣产品。

扎实办好民生实事。一是新增就业7.5万人，扶持创业5000人，带动就业2万人，发放小额担保贷款2亿元，免费培训新型农民4000人。二是开工建设保障性住房37937套，基本建成17981套，分配入住12332套。三是解决40万农村人口饮水安全问题。四是推进义务教育学校标准化建设。五是建设公安交通指挥系统，新增中心城区公交车100台，新建公交智能电子站台94座。六是淘汰中心城区98家企事业单位燃煤锅炉。七是为困难群众和农民工办理法律援助案件2400件，办理法律援助事项2.4万件以上。八是免费为100名贫困下肢残疾人安装假肢，为300名贫困白内障患者实施复明手术，为300名0至6岁贫困残疾儿童实施抢救性康复，为4000名贫困残疾人配发辅助器具。九是创建12个省级绿色示范乡村，新建75家放心粮油连锁店。十是实施中心城区背街小巷亮化工程，新建（更换）路灯1800盏，完成广播电视“户户通”8603户。对上述事项，我们将兑现承诺、一抓到底，努力把实事落实、好事办好。

三、运用法治方式推进政府自身建设

深入贯彻依法治国基本方略，严格依法行政、依法办事，加快建设职能科学、权责法定、执法严明、公开公正、廉洁高效、守法诚信的法治政府。

（一）强化法治思维，坚持依法行政。把公众参与、专家论证、风险评估、合法性审查和集体讨论决定作为重大决策的必经程序，减少决策失误。加强规范性文件管理，严格行政执法程序、执法标准和执法监督。认真执行人大及其常委会的决议和决定，积极支持人民政协发挥协商民主作用，主动听取民主党派、工商联、无党派人士和人民团体的意见和建议。自觉接受人大法律监督、工作监督和政协民主监督，认真办理人大代表建议和政协提案。深入开展法制宣传教育，建立法律顾问和培训制度，提高政府工作人员运用法治思维和法治方式解决问题的能力。

（二）强化责任担当，坚持高效施政。始终把务实高效作为政府工作的自觉追求，苦干实干、勇于担当，把改革发展的各项任务一件件落到实处。加强公务员队伍建设，提高公务员整体素质。积极推进政府绩效管理，用绩效引领工作导向、评定工作实绩、检验工作成果。加强督查考核，狠抓执行落实，确保政令畅通。强化治庸问责，坚决整治“中梗阻”、不作为、慢作为、乱作为，坚决克服懒政、怠政，坚决惩处失职、渎职。

（三）强化政务公开，坚持阳光理政。以公开为原则、不公开为例外，建立健全政务公开制度，加强对权力运行的制约和监督，让人民监督权力，让权力在阳光下运行。创新公开方式，扩大公开内容，努力做到决策、管理、服务、结果公开。加强政务电子建设，健全突发事件、公共危机事件和社会热点问题信息发布机制，不断增强政府工作的透明度。支持新闻媒体监督行政行为，积极回应网络舆论监督。

（四）强化廉政建设，坚持廉洁从政。认真落实“一岗双责”，建立健全教育、制度、监督并重的惩治和预防腐败体系。深入开展理想信念教育、岗位廉政教育，筑牢拒腐防变的思想防线。巩固和扩大党的群众路线教育实践活动成果，建立健全反“四风”、转作风的长效机制，坚持勤俭办事，反对铺张浪费。加强审计监督和行政审批、公共资源交易等重点领域和关键环节的监督，促进规范管理。加强行政监察，纠正行业不正之风，严肃查处腐败案件，以反腐倡廉的实际成效取信于民。

各位代表！振兴使命激励我们砥砺奋进，人民重托鞭策我们勇毅笃行。让我们紧密团结在以习近平同志为总书记的党中央周围，在中共荆州市委的坚强领导下，凝神聚力、鼓足干劲、克难攻坚，为实施壮腰工程、加快荆州振兴而努力奋斗！
